# Efficiency of Bone Marrow-Derived Mesenchymal Stem Cells and Hesperetin in the Treatment of Streptozotocin-Induced Type 1 Diabetes in Wistar Rats

**DOI:** 10.3390/ph16060859

**Published:** 2023-06-08

**Authors:** Osama M. Ahmed, Ablaa S. Saleh, Eman A. Ahmed, Mohammed M. Ghoneim, Hasnaa Ali Ebrahim, Mohamed A. Abdelgawad, Mohammed Abdel-Gabbar

**Affiliations:** 1 Physiology Division, Zoology Department, Faculty of Science, Beni-Suef University, P.O. Box 62521, Beni-Suef 62521, Egypt; osamamoha@yahoo.com; 2 Experimental Obesity and Diabetes Research Lab (EODRL), Zoology Department, Faculty of Science, Beni-Suef University, Beni-Suef 62521, Egypt; 3 Biochemistry Department, Faculty of Science, Beni-Suef University, P.O. Box 62521, Beni-Suef 62521, Egypt; 4 Department of Pharmacy Practice, College of Pharmacy, AlMaarefa University, Ad Diriyah, Riyadh 13713, Saudi Arabia; 5 Pharmacognosy and Medicinal Plants Department, Faculty of Pharmacy, Al-Azhar University, Cairo 11884, Egypt; 6 Department of Basic Medical Sciences, College of Medicine, Princess Nourah bint Abdulrahman University, P.O. Box 84428, Riyadh 11671, Saudi Arabia; 7 Department of Pharmaceutical Chemistry, College of Pharmacy, Jouf University, Sakaka 72341, Saudi Arabia

**Keywords:** bone marrow-derived mesenchymal stem cells, hesperetin, STZ diabetes

## Abstract

Type 1 diabetes mellitus (T1DM) was established to be ameliorated by islet transplantation, but the shortage of the transplanted human islet tissue and the use of immunosuppressive drugs to inhibit the rejection of allogeneic grafts make this type of therapy is limited. Nowadays, therapy with stem cells is one of the most promising future treatments. This kind of therapy could have a profound impact on both replacement, as well as regenerative therapies, to improve or even cure various disorders, including diabetes mellitus. Flavonoids have also been shown to possess anti-diabetic effects. Thus, this study aims to evaluate the effectiveness of the bone marrow-derived mesenchymal stem cells (BM-MSCs) and hesperetin in the treatment of a T1DM rat model. T1DM was induced in male Wistar rats that had been starved for 16 h via intraperitoneal injection of STZ at a dose of 40 mg/kg body weight (b.wt.). After 10 days of STZ injection, the diabetic rats were allocated into four groups. The first diabetic animal group was considered a diabetic control, while the other three diabetic animal groups were treated for six weeks, respectively, with hesperetin (given orally at a dose of 20 mg/kg b.wt.), BM-MSCs (injected intravenously at a dose of 1 × 10^6^ cells/rat/week), and their combination (hesperetin and BM-MSCs). The use of hesperetin and BM-MSCs in the treatment of STZ-induced diabetic animals significantly improved the glycemic state, serum fructosamine, insulin and C-peptide levels, liver glycogen content, glycogen phosphorylase, glucose-6-phosphatase activities, hepatic oxidative stress, and mRNA expressions of NF-κB, IL-1β, IL-10, P53, and Bcl-2 in pancreatic tissue. The study suggested the therapy with both hesperetin and BM-MSCs produced marked antihyperglycemic effects, which may be mediated via their potencies to ameliorate pancreatic islet architecture and insulin secretory response, as well as to decrease hepatic glucose output in diabetic animals. The improvement effects of hesperetin and BM-MSCs on the pancreatic islets of diabetic rats may be mediated via their antioxidant, anti-inflammatory, and antiapoptotic actions.

## 1. Introduction

Diabetes mellitus (DM) has become a major public health issue, affecting 12% of the global population. It is a metabolic illness with many etiologies that is ranked as the seventh largest cause of mortality worldwide [1,2]. Type 1 DM (T1DM) is an autoimmune illness characterized by an absolute deficiency of insulin and destruction of β-cells [3]. Damage of β-cells in the pancreas could result in a blood insulin regulation defect with elevated blood glucose levels, causing a deleterious carbohydrate, protein, and lipid metabolic disturbances, which could lead to the development of various complications in various organs, such as neuropathy, nephropathy, and retinopathy [4]. Continuous exogenous insulin therapy is the most common therapeutic technique employed in such patients and cannot be avoided; nonetheless, it cannot compensate for the normal β-cells’ sensitive adjustment [5].

Treatment of T1DM includes control of food intake and insulin injection, which cannot simulate the normal endogenous secretion of insulin in the body, leading to life-threatening complications, such as severe hyperglycemic attacks or hypoglycemic shocks [6]. In some circumstances, insulin therapy may fail to control the evolution of diabetes and its consequences in some circumstances. Therefore, various alternative therapies may be preferable. Another strategy of therapy depended on transplantation of the whole pancreas or pancreatic islet cells [7]. However, it was limited due to the shortness of the donors and rejection complications [8]. So, the ideal therapeutic tool would be the induction of the regeneration of the endogenous β-cells [9]. As a result, new therapy options are being investigated.

Older traditional treatments concentrated solely on insulin level regulation without addressing diabetes complications, but newer therapeutic approaches not only alleviate symptoms, but also improve organ function. Hence, a treatment strategy that endeavours to prevent the autoimmune damage of remaining and newly generated cells and to recover the lost insulin-secreting cells is highly desirable [10]. For being more targeted and specific therapeutic approaches, stem cell-based strategies illuminated new horizons for treating T1DM [11]. Consequently, interest in stem cells’ great regenerative capacity and differentiation potential attracted more attention from researchers [12,13,14].

Mesenchymal stem cells (MSCs) are considered one of the most promising types of stem cells for translational application because of their low immunogenicity, their rich tissue sources (bone marrow, liver, kidney, adipose tissue, urine, umbilical cord blood, and umbilical tissue), their multipotency, easy multiplication in vitro, and their ability to differentiate into specialized cells to repopulate injured tissues [15]. Principally, MSCs derived from bone marrow (BM-MSCs) have become one of the research focusses of cell therapy for diabetes, ischemic diseases, and neurologic disorders [16]. They are one of the earliest progenitors of stem cells to be inserted in the clinic because they reproduce widely [17], they are easy to isolate and obtain by clinical procedures based on the ability of adherence to plastic surfaces [18], and they have the potential to differentiate into multiple cell types. In our previous study, it was found that BM-MSCs alone and, in combination with the flavonoid chrysin, enhanced oral glucose tolerance via lowering inflammation, restoring β-cell functionality, and attenuating insulin resistance; the combination was the most effective treatment [19].

Hesperetin is a citrus flavonoid that has lately gained attention in scientific investigations due to its characteristics in fighting DM and its associated consequences. It has been extensively studied for its potential antihyperglycemic, hypolipidemic, and anti-cancer properties [20,21,22,23]. Researchers have discovered that hesperetin has a putative antioxidant defense-related enzyme activity and, hence, acts as a cell’s radical scavenger and promoter [24,25]. Further, it has been described as possessing powerful properties against inflammation [26]. Mainly, among other flavonoids, we targeted hesperetin, which is simply and passively absorbed across the intestine into the circulation and is excreted in the urine within 24 h of its consumption [27], which may enhance its bioactivity and effectiveness. In our previous study, the use of hesperetin in combination with BM-MSCs enhanced their improvement effects on lipid profiles and on heart and kidney functions in diabetic rats [28].

As a result, we conducted a study to assess the in vivo antihyperglycemic effects and suggest the mechanisms of action of hesperetin, BM-MSCs, and their combination in STZ-induced diabetic rats by measuring serum insulin, C-peptide, and fructosamine levels, as well as liver glycogen content, the activities of glycogen phosphorylase (GP) and glucose-6-phosphatase (G6Pase) enzymes, in addition to hepatic oxidative stress and antioxidants, inflammatory modulators, such as nuclear factor-kappa B (NF-κB), interleukin-10 (IL-10), interleukin-1β (IL-1β), tumor necrosis factor-α (TNF-α), interleukin-17 (IL-17), and interleukin-4 (IL-4), and expression of genes related to apoptosis, such as genes of protein 53 (P53) and B-cell lymphoma 2 (Bcl-2).

## 2. Results

### 2.1. Fasting and 2-h Post-Prandial Glucose Levels at Pre- and Post-Treatment Periods

As shown in Table 1, surveying blood glucose levels at 10 days after STZ injection indicated that the diabetic groups exhibited no significant difference in blood glucose levels at fasting state and at 2 h post glucose loading. The diabetic control group showed marked further deteriorations in blood glucose levels as the period extended to the end of the experiment. On the other hand, the diabetic groups treated with hesperetin and BM-MSCs exhibited a significant decrease (*p* < 0.05) in blood glucose levels at fasting state and 2 h post glucose loading as compared with that of the diabetic control group at the end of the experiment; the combinatory effect of hesperetin and BM-MSCs seemed to be more potent.

### 2.2. Evaluation of OGTT

**Figure 1:**
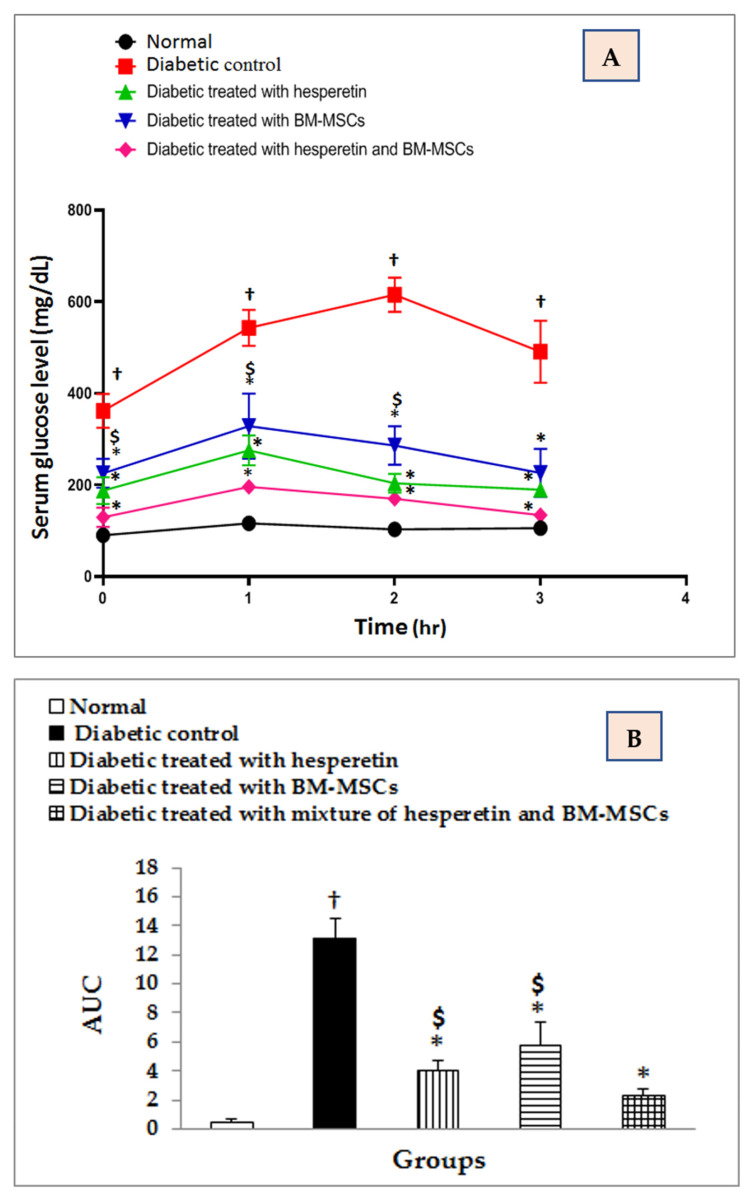
Oral glucose tolerance curves (**A**) and AUC (**B**) of normal, diabetic control, and diabetic groups treated with hesperetin, BM-MSCs, and their combination for six weeks. ^†^ *p* < 0.05 compared with normal group; * *p* < 0.05 compared with diabetic group; ^$^ *p* < 0.05 compared with diabetic group treated with the combination of hesperetin and BM-MSCs.

The OGTT data (Figure 1A) and area under curve (Figure 1B) of the normal, diabetic control and diabetic treated groups were shown. The serum glucose levels at all intervals (0, 1, 2, 3 h) were significantly (*p* < 0.05) increased in diabetic group in comparison with the normal. However, the treatment with hesperetin, BM-MSCs, and their combination effectively improved (*p* < 0.05) the impaired glucose tolerance of diabetic rats, and the combination of hesperetin and BM-MSCs appeared to be the most effective. AUC exhibited a significant increase (*p* < 0.05) in the diabetic control. The treatment with hesperetin, BM-MSCs, and their combination significantly decreased (*p* < 0.05) AUC; the combinatory effects was the most potent. The AUC was significantly lower in the diabetic group, with the combination, when compared with single treatments.

### 2.3. Effects on Serum Fructosamine Level

The serum fructosamine concentration showed a significant (*p* < 0.05) increase in diabetic rats as compared to normal control. Treating the diabetic rats for six weeks with hesperetin, BM-MSCs, and their combination motivated a significant decrease in serum fructosamine concentration comparing to the diabetic control (Table 2). Though, there was no significant variance between the effects of hesperetin, BM-MSCs, and their combination on serum fructosamine concentration.

### 2.4. Effects on Serum Insulin and C-Peptide Levels

As shown in Table 2, the levels of insulin and C-peptide revealed a significant (*p* < 0.05) decrease in diabetic rats with percentage changes of −51.6% and −59.04% with respect to comparisons with normal control rats. All of the therapies supplied to diabetic rats for six weeks significantly (*p* < 0.05) increased insulin levels. Principally, hesperetin appeared to be the most effective in enhancing insulin level of diabetic rats. Similarly, all the treatments succeed to increase C-peptide levels significantly (*p* < 0.05), and the combinatory group was significantly more potent when compared to treatment with BM-MSCs alone.

### 2.5. Effects on Hepatic Glycogen Levels

The liver glycogen level displayed a significant (*p* < 0.05) decrease in diabetic rats compared to normal control rats. Conversely, diabetic rats treated with hesperetin, BM-MSCs, and their combination showed significant elevation of hepatic glycogen level (Table 3). BM-MSCs appeared to be more effective in ameliorating hepatic glycogen levels in diabetic rats.

### 2.6. Effects on Hepatic G6Pase and GP Levels

Hepatic level of G6Pase and GP increased significantly (*p* < 0.05) in diabetic rats compared to the normal control. The supplementation for six weeks with hesperetin, BM-MSCs, and their combination significantly (*p* < 0.05) decreased hepatic levels of G6Pase and GP. Hesperetin and BM-MSCs seemed to be the most effective in improving hepatic levels of G6Pase and GP than their combination. ANOVA analysis revealed that the effect between groups on hepatic glycogen, G6Pase, and GP levels was significant (*p*< 0.05) throughout the experiment, as illustrated in Table 3. The diabetic group treated with the mixture was significantly more potent in decreasing GP activity than that treated with hesperetin alone.

### 2.7. Effects on Hepatic LPO and GSH and GPx Activities

As illustrated in Table 4, the malondialdehyde (MDA), as a biomarker of lipid peroxidation (LPO), was significantly (*p* < 0.05) higher in the liver of the diabetic group than those in the normal control one. Whereas, when providing the STZ-diabetic rats with hesperetin, BM-MSCs and their combination prevented this elevation to a great extent (*p* < 0.05) (Table 4). BM-MSCs and combination treatment of hesperetin and BM-MSCs appeared to be the most potent for improving hepatic MDA level. Additionally, the liver GSH and GPx activities were significantly (*p* < 0.05) decreased in diabetic rats, as compared to normal control group. The diabetic rats supplied with hesperetin, BM-MSCs, and their combination significantly (*p* < 0.05) ameliorated the decreased GSH and GPx activities. The effect of BM-MSCs and the concurrent treatment with hesperetin and BM-MSCs seemed to be the most potent on reducing liver LPO and enhancing liver GPx activity. The effect of BM-MSCs seemed to be the most efficient on increasing liver GSH content.

### 2.8. Effects on Hepatic GST and GR Activities

The liver GST and GR activities were significantly (*p* < 0.05) decreased in rats with STZ-induced diabetes when compared to normal control group. The STZ-induced diabetic rats administered with hesperetin, BM-MSCs, and their combination significantly ameliorated (*p* < 0.05) the depleted GST and GR activities, and hesperetin seemed to have more potential for increasing GST activity. Furthermore, the hesperetin and BM-MSCs had potential for increasing GR activity. However, there was no significant difference between all the treated groups regarding GR activity. Regarding one-way ANOVA, it was found that the variance between groups on GST and GR activities was significant (*p* < 0.05), as showed in Table 5.

### 2.9. Effects on mRNA Levels of NF-κB, IL-1β and IL-10

As expressed in Table 6, the STZ-administered rats showed a significant (*p* < 0.05) increase in pancreatic NF-κB and IL-1β levels, as compared to normal control rats. The supplementations of hesperetin, BM-MSCs, and their combination potentially decreased (*p* < 0.05) the altered levels of NF-κB and IL-1β relative to normal control rats. BM-MSCs and hesperetin treatment successfully diminished the NF-κB levels than their combination, while the combinatory group was effective with regards to decreasing IL-1β levels. The IL-10 mRNA expression level exhibited an opposite behavioral pattern; its expression was down-regulated significantly (*p* < 0.05) in STZ-administered rats, as compared to normal control rats. The treatment of STZ-administered rats with hesperetin, BM-MSCs, and their combination produced a significant increase (*p* < 0.05) in the decreased IL-10 mRNA level. The treatment with BM-MSCs and the combinatory group were more effective in increasing IL-10 mRNA expression than the treatment with hesperetin alone (Table 6).

### 2.10. Effects on Serum TNF-α, IL-17 and IL-4 Levels

The STZ-induced diabetic rats exhibited a significant increase (*p* < 0.05) in serum TNF-α and IL-17 levels and a significant decrease in serum IL-4 level. The treatment of STZ-administered rats with hesperetin, BM-MSCs, and their combination produced a significant amelioration (*p* < 0.05) in these alterations. The combinatory effects of hesperetin and BM-MSCs on IL-17 level was significantly more potent (*p* < 0.05) than the effects of hesperetin and BM-MSCs. The combination was significantly more effective (*p* < 0.05) than hesperetin regarding decreasing the elevated TNF-α serum levels (Table 7).

### 2.11. Effects on mRNA Levels of P53 and Bcl-2

Compared to the normal control pancreas, the diabetic control animals caused a remarkable (*p* < 0.05) upregulation in P53 level in the pancreas, in contrast to the down-regulated level of Bcl-2. Conversely, the treatment of diabetic rats with hesperetin, BM-MSCs, and their combination treatments inhibited activation of P53 and induced activation of Bcl-2 mRNA expression. The effect of the mixture of hesperetin and BM-MSCs seemed to be the most potent with regards to decreasing P53 and increasing Bcl-2 (Table 8). The combinatory effect of hesperetin and BM-MSCs was more significantly potent than single treatment with either hesperetin or BM-MSCs.

### 2.12. Histopathological Changes

**Figure 2:**
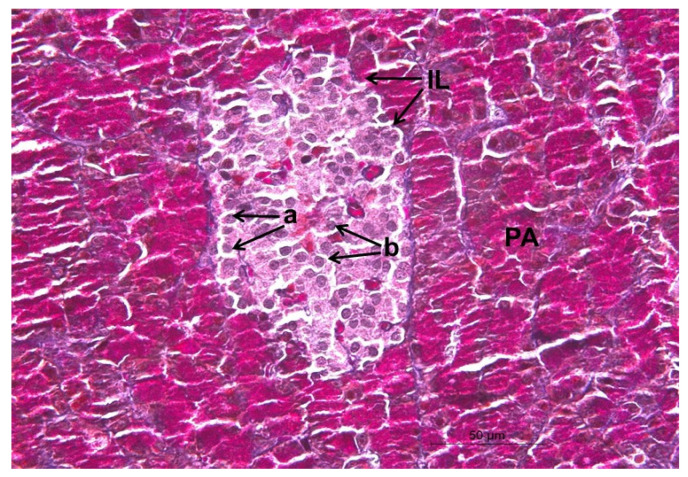
Photomicrograph of a section of a pancreas of a normal rat, showing their histological structure with closely packed lobules of pancreatic acini (PA) and intact β-cells (b) of Langerhans islets (IL) embedded within the exocrine portions and α-cells (a).

**Figure 3:**
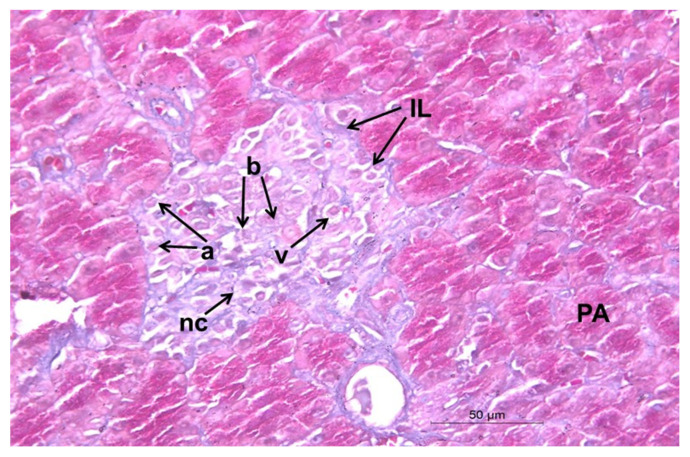
Photomicrograph of a section of a pancreas of a diabetic rat, showing histopathological changes in endocrine portions represented by a marked decrease in β-cells (b) and obvious vacuolation (v) of β-cells and necrosis (nc). IL, PA and a refer to islets of Langerhans, pancreatic acini and α-cells, respectively.

**Figure 4:**
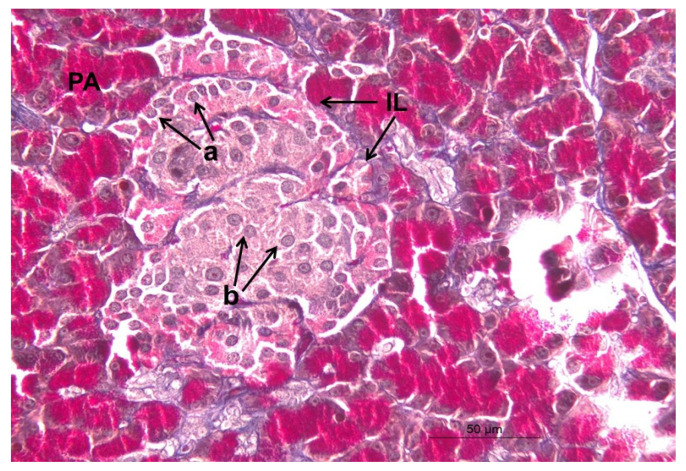
Photomicrograph of a section of a pancreas of a diabetic rat treated with hesperetin, showing their histological structure with increases in β-cells number through replication of β-cells and progenitor cells, and decreases in vacuolation were detected. IL, PA, a and b refer to islets of Langerhans, pancreatic acini, α-cells and β-cells, respectively.

**Figure 5:**
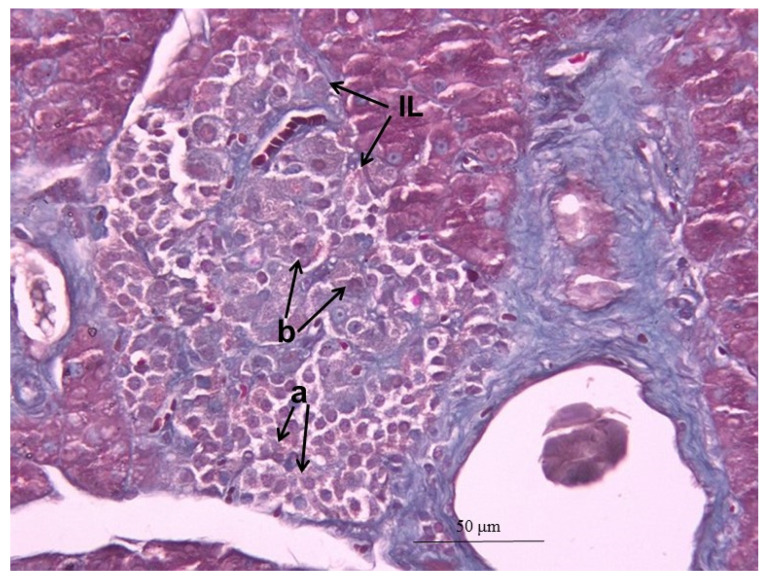
Photomicrograph of section of a pancreas of a diabetic rat treated with BM-MSCs showing their histological structure with a marked increase in β-cell (b) number and increase in the size of the islets of Langerhans (IL). The symbol “a” refer to α-cells.

**Figure 6:**
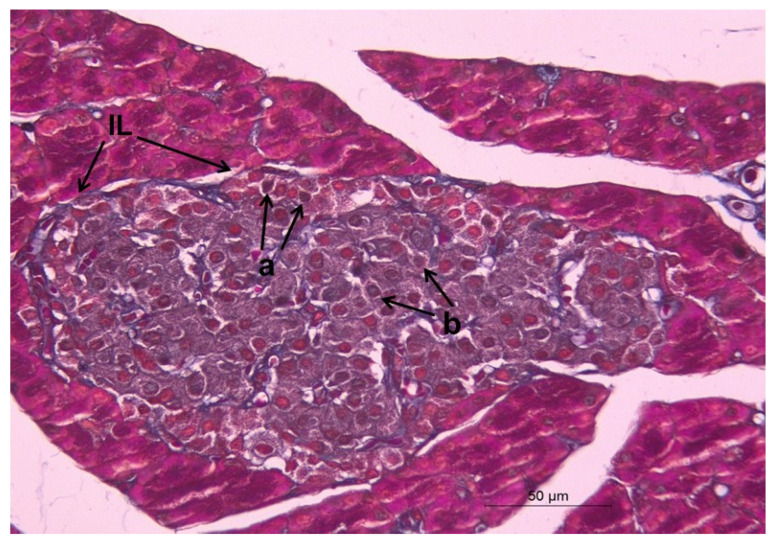
Photomicrograph of a section of the pancreas of a diabetic rat treated with the mixture of hesperetin and BM-MSCs, showing a greater improvement in the pancreatic histological changes than those of rats treated with hesperetin or BM-MSCs. IL, a and b refer to islets of Langerhans, α-cells and β-cells, respectively.

The histopathological findings in pancreas sections from the five experimental groups are depicted in Figure 2, Figure 3, Figure 4, Figure 5 and Figure 6, (Trichrome PAS stain; Scale bar = 50 µm). The pancreatic sections of normal control rats showed normal lobules with well and closely packed pancreatic acini, as well as intact β-cells of Langerhans islets implanted within the exocrine portions and alpha cells (Figure 2). By contrast, diabetic control rats indicated histopathological alterations in the endocrine portion established by a considerable decrease in β-cells and obvious vacuolation and necrosis of β-cells (Figure 3). These variations were rather modified in STZ-administered rats treated with hesperetin via replication of β-cells and progenitor cells and decline of vacuolation, as detected in Figure 4. The medication of the animals with BM-MSCs produced mild improvement in the pancreatic histological changes, as compared to STZ-administered rats. There was a marked increase in β-cell number, but this was still not efficient for insulin secretion (Figure 5). Moreover, providing the diabetic rats with hesperetin and BM-MSCs produced an obvious endocrine cell proliferation, as well as marked improvement of the pancreatic histological architecture compared to STZ-control rats, as illustrated in Figure 6. Table 9 depicted the histopathological lesion score of islet cells’ vacuolations and necrosis and revealed severe lesion score (+++) in diabetic rats. These lesion scores were decreased in the diabetic rats treated with hesperetin (+, −), BM-MSCs (++, +), and their combination (−, −), respectively.

### 2.13. Immunohistochemical Staining of Insulin

**Figure 7:**
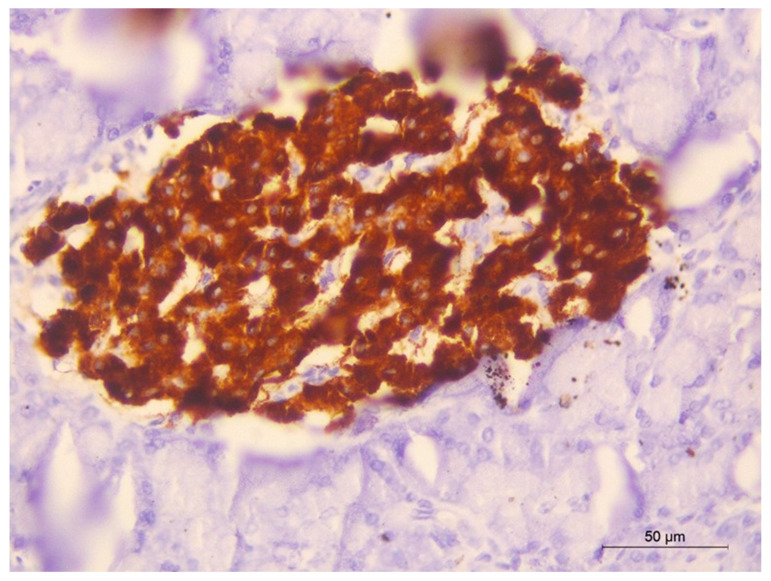
Photomicrograph of sections of a pancreas of a normal rat, showing its strong positive reaction for anti-insulin antibodies as brown granules in the cytoplasm of a great number of the β-cells.

**Figure 8:**
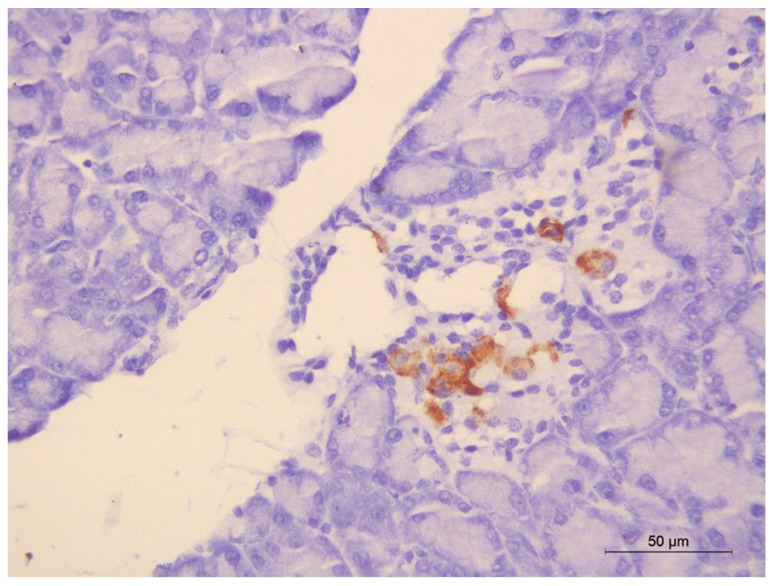
Photomicrograph of a section of a pancreas of a diabetic rat, showing that the immune reactivity for anti-insulin antibodies was obviously decreased in the cytoplasm of a great number of the β-cells.

**Figure 9:**
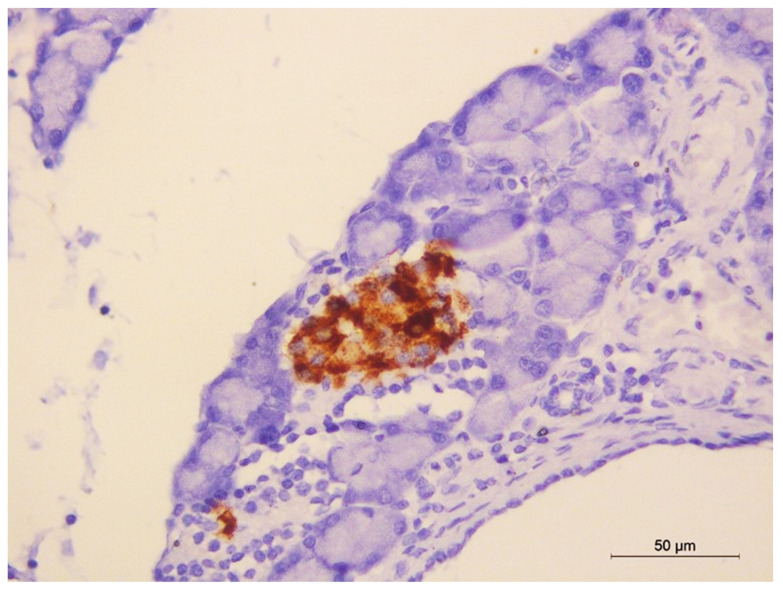
Photomicrograph of a section of a pancreas of a rat treated with hesperetin, showing increased positive immunoreactions of β-cells for anti-insulin antibodies.

**Figure 10:**
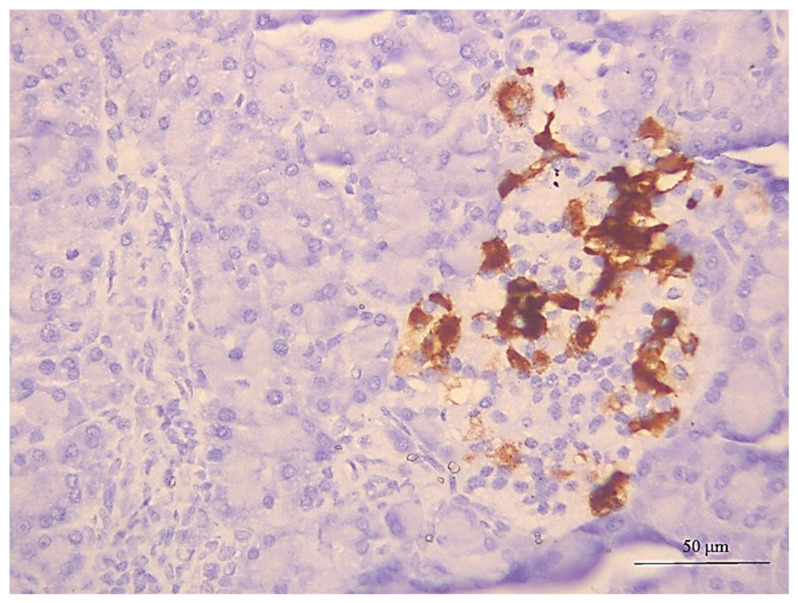
Photomicrograph of a section of a pancreas of a rat treated with BM-MSCs, showing increased positive immunoreactions of β-cells for anti-insulin antibodies. However, many β-cells are still insufficient for insulin secretion.

**Figure 11:**
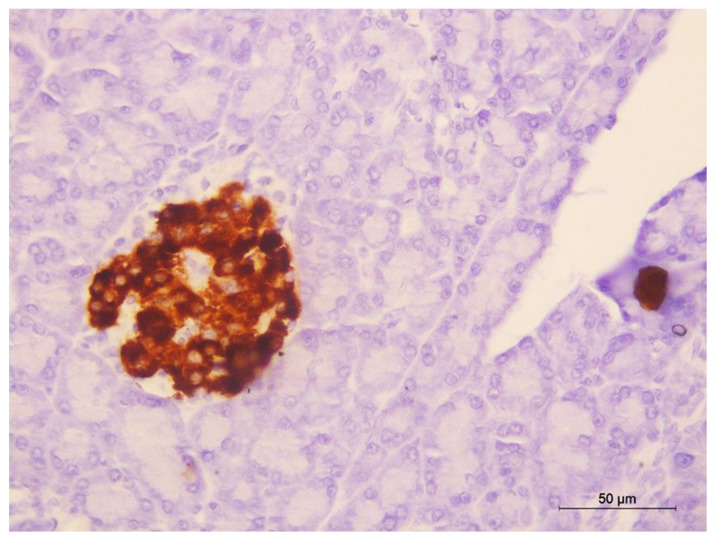
Photomicrographs of a section of a pancreas of a rat treated with a mixture of hesperetin and BM-MSCs, showing markedly increased positive immunoreactions of β-cells for anti-insulin antibodies.

**Figure 12:**
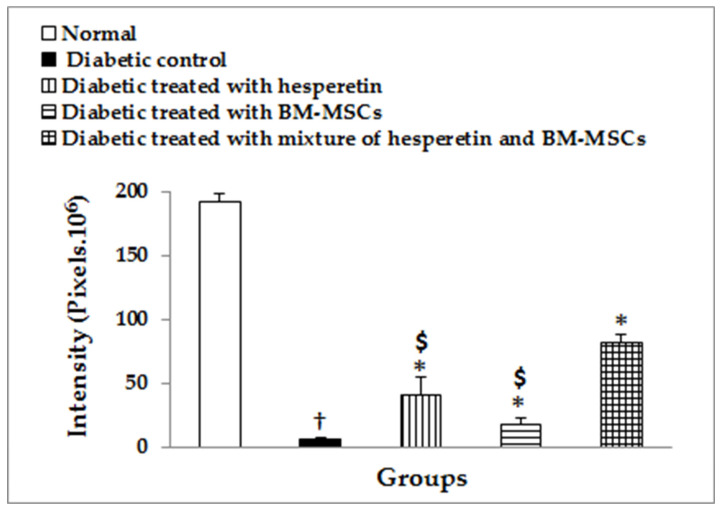
Effect of hesperetin and BM-MSCs on brown stain intensity of the islets of Langerhans of pancreases in STZ-induced diabetic rats. ^†^ *p* < 0.05 compared with normal group; * *p* < 0.05 compared with diabetic control group; ^$^ *p* < 0.05 compared with diabetic group treated with the combination of hesperetin and BM-MSCs.

Immunohistochemically, β-cells from the normal group showed a robust positive reactivity to anti-insulin antibodies as brown granules in the cytoplasm of a large number of β-cells (Figure 7). In the diabetic group, the immunological reactions for anti-insulin antibodies was obviously reduced (Figure 8). On the other side, positive immunoreactions of β-cells to anti-insulin antibodies were clearly elevated following hesperetin (Figure 9), as well as BM-MSCs, therapies (Figure 10). Besides, treatment with hesperetin and BM-MSCs resulted in much higher positive immunoreactions (Figure 11). Image analysis in Figure 12 revealed a significant decrease (*p* < 0.05) in brown colour intensity in the diabetic rats. The treatment of diabetic rats with hesperetin, BM-MSCs, and their combination significantly increased (*p* < 0.05) the brown colour intensity, reflecting a significant increase in insulin expression in the β-cells; the combinatory effect was the most potent.

## 3. Discussion

DM is a complex metabolic condition characterized by chronic hyperglycemia and disruptions in carbohydrate, lipid, and protein metabolism caused by flaws in insulin secretion, action, or both [29]. It is one of the most common metabolic illnesses and is regarded as one of the primary causes of death worldwide [30]. The disease is complex, and it has a substantial influence on patients’ health, quality of life, and life expectancy, as well as the healthcare system [31]. Chronic and persistent hyperglycemia in DM is linked to long-term damage, dysfunction, and failure of multiple organs, particularly the eyes, kidneys, nerves, heart, and blood vessels [32]. Impairment of growth and susceptibility to certain infections may also accompany chronic hyperglycemia. Diabetes-related mortality and morbidity are primarily attributable to comorbidities, such as neuropathy, nephropathy, vasculopathy, and retinopathy [33].

According to previous research, hyperglycemia can be treated by transplanting pancreatic or islet cells to replace cells that have lost function and, therefore, reduce the need for insulin [34]. However, the shortage in human islet tissue and the usage of immunosuppressive drugs to prevent rejection of allogeneic grafts limit the use of such therapies in type 1 diabetic patients [35].

Hesperetin is one of the most important citrus flavonoids that has been proven to have antioxidant and anti-inflammatory characteristics, as well as anti-apoptotic capabilities [22]. Additionally, preclinical and clinical evidence has provided a promising therapeutic benefit of MSCs in various medical conditions due to their regenerative benefits and ease of separation. Furthermore, they are less immunogenic due to the intermediate expression of major histocompatibility complex (MHC) class I, as well as the absence of MHC class II and costimulatory molecules on their cell surfaces [36]. Besides, MSCs release a multitude of cytokines, growth factors, and exosomes that play an important role in the regulation of insulin sensitivity and β-cell dysfunction [37]. In our present work, we isolated BM-MSCs from adult male Wistar rats. MSCs were described by their adhesiveness and fusiform shape, which is in accordance with the findings of El Barky et al. [38]. During the change in medium, MSCs preserved their adherence in culture, while all of the other nonadherent cells were removed by washing. Moreover, the ability of MSCs to adhere in culture flasks is enhanced by the selective medium and the polystyrene-coated tissue culture flasks [39].

OGTT is an assay that is well accepted and frequently utilized to screen the antihyperglycemic potential of any hypoglycemic agent [40]. The present data elucidate a significant elevation in levels of serum glucose in diabetic rats in comparison with the normal controls. These results are in harmony with the study of El Barky et al. [38] and Ahmed et al. [41] and could be attributed to beta-cell impairment, resulting in a significant reduction in insulin manufacturing, thus establishing hyperglycemia syndrome. Moreover, reports by Gad and El-Maddawy [42], as well as Kamal [43], explained that glucose intolerance might be caused by either a defect in secretion of insulin (T1DM disorder) or a defect in insulin sensitivity (T2DM disorder). However, our findings clarified that the diabetic rats treated with hesperetin, BM-MSCs, and their combination exhibited an obvious decrease in high serum glucose levels.

In our present research, oral administration of hesperetin every day for six weeks induced a significant decrease in concentrations of serum glucose and improvement in the oral glucose tolerance and AUC in STZ-induced DM. These results are in parallel with Revathy and Sheik Abdullah [33], who observed that oral injection of hesperetin for six weeks stimulated a substantial decrease in concentrations of plasma glucose in diabetic animals. According to our data, hesperetin as a therapeutic agent showed an antihyperglycemic potential, and this may be due to enhancement in the insulin secretion from the existing β-cells and regenerated pancreatic β-cells. The increase in serum insulin and C-peptide levels, in addition to improvement in pancreatic islets’ architecture and structural integrity, support this attribution.

Our study demonstrated that allogenic MSC intravenous implantation significantly improved the glycemic state in Wistar rats with STZ-induced diabetes. This was indicated by improvements in oral glucose tolerance and AUC by treatment of diabetic rats with BM-MSCs; the effect was more enhanced by co-treatment with hesperetin. These findings are consistent with those of Bhansali et al. [44] and Hess et al. [45], who found a significant reduction in blood glucose levels four days after bone marrow-derived cell transplantation into STZ-treated mice, possibly due to the activation of endogenous cells to proliferate and produce insulin. Furthermore, Bell et al. [46] ascribed MSCs’ antihyperglycemic effect to islet repair via direct differentiation into functional β-cells. Additionally, Dong et al. [47] described that MSCs were homed to the pancreas of recipients and transdifferentiated into insulin-generating cells. The increase in the serum insulin and C-peptide levels and increase in the size of islets of Langerhans and number of β-cells within the islets support the crucial role of insulin secretion in controlling the glycemic state in the diabetic rats treated with BM-MSCs.

Many authors have proposed serum fructosamine, a glycosylated protein measuring technique, as a valuable tool for DM screening [48,49]. In our present work, serum fructosamine level was significantly improved in fasting diabetic rats in contrast to normal ones. These results are consistent with Ahmed et al. [50]. The significant increase in hexosamine level in the tissues and plasma of diabetic rats, which may have occurred by deficiency of insulin, suppresses glucose utilization via an insulin-dependent pathway, thus improving the development of both hexose and hexosamine [51]. However, the diabetic rats treated with hesperetin, BM-MSCs, and their combination exhibited an obvious decline in its elevated level. These results agree with Revathy and Sheik Abdullah [52], as well as Farid et al. [53].

In our study, we observed low levels of plasma insulin and C-peptide in diabetic rats. Such results are in harmony with Jasminenathesha et al. [54]. Additionally, Akhani et al. [55] and Ahmed [56] illustrated that fasting insulin level significantly decreased in STZ diabetic rats and may be attributed to the diabetogenic effect of STZ, which causes damage and reduces the number of pancreatic β-cells in the Langerhans islets, as occurred in the current study. On the other hand, the treatment of STZ-induced diabetic rats with hesperetin, BM-MSCs, and their combination significantly improved the fasting insulin and C-peptide levels. These results also run parallel with Mahmoud et al.’s [57] study, wherein they claimed that the administration of a hesperetin analog, hesperidin, to diabetic rats, resulted in a remarkable increase in serum insulin levels, and this was qualified to the protective actions of β-cells and their promoting effects on the insulin secretory response of Langerhans islets, as elucidated by the in vivo study. Kappel et al. [58] confirmed that flavonoids improve intracellular concentration of Ca^2+^ and induce ATP-sensitive K+ channels of pancreatic islets’, a preparatory step in the synthesis of insulin to be suppressed. Overall, hesperetin can boost insulin secretion by promoting the survival of pancreatic islet cells. Systemic administration of BM-MSCs gives rise to a recovery of pancreatic islets, enhances secretion of blood insulin, and corrects hyperglycemia, as reported by Jurewicz et al. [59] and Ezquer et al. [60].

Hepatic glycogen level may be the strongest predictor of any drug’s antihyperglycemic ability [61]. Our results showed a profound decrease in content of hepatic glycogen, followed by an enormous elevation in the activity of hepatic GP and the gluconeogenic enzyme, G6Pase, in diabetic control rats, as compared with that of normal control ones. These findings run parallel with those of Ahmed [62] and Abdel-Moneim et al. [63]. Besides, Raju et al. [64] stated that diabetes’s elevated hepatic glucose production is accompanied by glycogenolysis and gluconeogenesis. Additionally, Ahmed [65] depicted that these variations may be attributed to deficiency in insulin and resistance to insulin action, thus causing the stimulation of glycogenolytic and gluconeogenic processes. Furthermore, hepatic tyrosine kinase decreased when insulin was insufficient to activate glycogen synthase. This is why why glycogen content decreases in diabetic animals [66]. In parallel, Miyamoto and Amrein [67] accounted that elevated hepatic endogenous glucose production is caused by enhanced activities of gluconeogenic enzymes, and this results in chronic hyperglycemia, and this may be attributed to insulin deficiency in a diabetic state. On the other hand, treatment of diabetic rats with hesperetin and BM-MSCs enhanced these altered parameters. These results are consisting with Jayaraman et al. [22], who reported that glycogen content in hepatocytes is enhanced, and this might suggest increased insulin secretion from the remaining β-cells in the pancreatic islets, and it was reported that glucose metabolic enzymes in diabetic rats are ameliorated by hesperetin. Similarly, amelioration of liver glycogen content has been proven in STZ-diabetic rats when other citrus flavonoids, such as hesperidin, were supplied [68]. Additionally, Constantin et al. [69] stated that hesperetin suppressed the isolated perfused rat liver’s gluconeogenesis.

In the present work, the hesperetin and BM-MSC treated groups elucidated a major observable elevation in hepatic glycogen levels. Such effect could be explained by increased usage of glucose, increased activity of hepatic hexokinase, and decreased activity of G6Pase, following the treatment with hesperetin and BM-MSCs. Additionally, in vivo studies indicated that massive formation of glucose-6-phosphate resulted and lead to inhibition of GP and activation of glycogen synthase in the liver and muscle [70]. In our opinion, the increase in liver glycogen content in association with the decrease in GP and G6Pase activities by treatments of diabetic rats with hesperetin and BM-MSCs may result in a decrease in hepatic glucose production, which plays an important role in the control of blood glucose levels in the blood.

Oxidative stress is considered a state characterized by imbalance between oxidants and antioxidants, which results in potential cellular impairment. The utmost cells can overcome oxidative stress in a mild degree. The imbalance occurs in case of insufficiency of antioxidant capacity triggered by disruption in the manufacture and distribution, or by a plethora of ROS through other factors [71]. The liver is the focal organ of the detoxification processes, in addition to free radical reactions and the oxidative stress biomarkers elevated in the liver in numerous disorders involving DM [72]. Hyperglycemia in the experimental animal models of DM may cause hepatic oxidative stress, in addition to the toxic effects of STZ [73]. The insulin deficiency and hyperglycemia resulting from β-cell necrosis further motivate liver damage caused by the hepatic membrane’s LPO triggered by ROS [74].

LPO is a crucial biomarker of oxidative stress that received extreme focus [75]. LPO results in malondialdehyde (MDA) formation, which is considered as an indication of lipid peroxides level via reaction with thiobarbituric acid [76].

The present work observed elevation of MDA level in the livers of STZ-induced diabetic rats, which runs parallel with Rajasekaran et al. [77], who observed intense increase in the thiobarbituric reactive substances (TBARS) and hydroperoxides’ concentrations in the liver of diabetic rats. These findings are also in accordance with El Barky et al. [38]. Additionally, Ugwu et al. [78] reported that DM was induced by a single intraperitoneal dose of STZ in rats, causing a significant raised concentration of MDA level in liver. Conversely, treatment with BM-MSCs alone, or concurrently with hesperetin, dramatically reduced MDA levels in liver tissues. These findings are in accordance with Narenjkar et al. [79], who recorded a higher level of MDA in the liver and heart tissues, following diabetes induction, and this was markedly reduced after hesperetin treatment. These results are also in accordance with Fang et al. [80], who declared that the diabetic rats that were treated with autologous MSC transplants from adipose tissue exhibited a significant decrease in MDA as compared to the vehicle-treated and untreated diabetic animals.

In the current research work, the STZ-administered rats exhibited a decrease in the activities of enzymatic antioxidants (GR, GST, and GPx) and nonenzymatic antioxidants (GSH) in liver tissues, as compared to normal control rats. These findings are in accordance with Sheweita et al. [81], Gokce and Haznedaroglu [82], and Zhang et al. [83], who reported that the decreased activities of catalase, GPx, and hepatic GR of the diabetic group could be attributed to ROS generated by STZ. Similar results were reported by Zahran et al. [84], who stated that STZ-induced diabetic rats exhibited a marked decrease in enzymatic activity of GPx in liver. Additionally, the study by Jasminenathesha et al. [40] elucidated a marked decrease in hepatic GSH, GPx, and GR in STZ-induced diabetic animals. Moreover, Coskun et al. [85] explained the decrease in GSH level of diabetic rats in relation to its usage to relieve oxidative stress in diabetes.

GSH, along with GPx and GST enzymes, are responsible for converting H_2_O_2_ or other organic hydroperoxides into non-toxic substances [86]. Therefore, the depletion of GSH level in the livers of diabetic rats has been related to the decrease in the activities of GST, GPx, and GR, and it is also related to the build-up of oxidative stress products, such as LPO, protein oxidation products (POPs), and advanced glycation end-products (AGEs) [87,88]. Decreased GPx activities may be promoted by radical-induced suppression and glycation of the enzyme (Hodgson and Fridovich, 1975). Additionally, GSH insufficiency may potentially reduce GPx activity [89]. The decreased GST activity observed in the diabetic state may be attributed to ROS-induced inactivation [90]. On the other hand, treating the diabetic rats with hesperetin alleviated oxidative stress by increasing GSH level and inducing the activities of antioxidant enzymes GPx, GR, and GST, which were altered in diabetic rats. These results are consisted with Miyake et al. [91], who reported that the level of GSH for the diabetic rat groups administered lemon flavonoids was higher than for the diabetic rats. Similarly, Revathy and Sheik Abdullah [29] showed a substantial increase in GPx activity, which was observed in diabetic rats treated with hesperetin, which may be ascribed to its scavenging property.

Our study exhibited that BM-MSCs administered as a therapeutic agent for diabetic rats remarkably alleviated oxidative stress by increasing GSH level and stimulating the activities of enzymatic antioxidants (GPx, GR, and GST) that were altered in diabetic rats. These results run parallel with Jasminenathesha et al. [54], who showed that BM-MSCs normalize GPx and GSH levels in the diabetic groups, as they regenerate the destructed β-cells and improve the glycemic state.

There was a marked elevation in the enzymatic and also non-enzymatic antioxidant guards in the livers of rats cured with hesperetin, BM-MSCs, and their combination when compared to animals administered STZ alone. This increase is attributed to the efficiency of hesperetin, BM-MSCs, and their combination to prevent the free radical’s formation, thus improving the endogenous antioxidant activity beyond their capacity to scavenge free radicals and the lowering of hepatic lipoperoxide formation. The suppression of the oxidative stress and enhancement of the antioxidant defense system by treatment with hesperetin, BM-MSCs, and their combination could play a significant role in the amelioration of pancreatic islets histological integrity and function, resulting in an increase in blood insulin levels and, in turn, improvement in the glycemic state.

Oxidative stress can activate various transcription factors. Stimulation of these transcription factors results in the expression of many different genes, involving those for pro-inflammatory proteins [92]. NF-κB is an essential factor of the signaling pathways that induce transcriptional control of numerous inflammatory agents involving IL-1β and TNF-α [93]. NF-κB is activated by hyperglycemia, and it mediates inflammation by activating the expression of proinflammatory and inflammatory cytokines [94]. In the present study, following STZ injection, a marked inflammation of the pancreas occurred, which is characterized by the induction of the NF-κB signaling system and its downstream genes, as well as increased production of inflammatory factors (IL-1β and TNF-α). Such findings are in concurrence with those of Cipollone et al. [95], who elucidated the stimulation in the expression of NF-κB in patients with T1DM. In parallel, Cnop et al. [96] illustrated that the transcription factor NF-κB is involved in β-cell apoptosis moreso in Type 1 than Type 2 diabetes. Data of our research showed that the treatment with hesperetin and BM-MSCs enhanced the inactivation of NF-κB in STZ-administered rats. These data were approved by Maggini et al. [97], who reported inhibition of NF-κB by MSCs. Additionally, Yoshida et al. [98] reported that hesperetin, as a citrus flavonoid, suppress TNF-α induced a release of free fatty acids (FFA) from mouse adipocytes through stimulation of extracellular signal-regulated kinase (ERK) pathways.

IL-1β is the active cytokine that gives rise to suppression of more insulin production and less islet cell survival [99,100]. Cytokines play a selective damaging action on β-cell function, as IL-1β cannot reduce the α-cells’ capacity to metabolize glucose to CO_2_, as stated by Corbett et al. [101]. In our present study, IL-1β mRNA expression increased significantly in pancreatic tissues of rats with STZ-induced diabetes. These changes are in accordance with Xu et al. [102], who reported that hyperglycemia activates macrophages to improve the production of proinfammatory cytokines, such as IL-1β, TNF-α, IL-6, IL-18, IL-12, and IFN-γ, both in vivo and in vitro.

To the contrary, the outcomes of our investigation revealed that the diabetic rats treated with hesperetin and BM-MSCs exhibited a marked reduction in the pancreatic tissue mRNA expression of IL-1β compared to animals administered STZ alone. Likewise, an in vivo study by Mahmoud et al. [57] summarized that a hesperetin analog, hesperidin, can frustrate the suppressive effect stimulated by IL-1β concerning the functioning of islet β-cells. Similar results are also in agreement with Benavente Garcia and Castillo [103] and Sridharan et al. [104], who reported that citrus flavonoids repress the formation of proinflammatory interleukins and cytokines, mainly TNF-α, as well as IL-1β, directly. These results are also consistent with Zhao et al. [105], who established that pancreatic pathological signs of necrosis and inflammation were diminished along with the decline of IL-1β mRNA expression as a result of MSCs transplantation in both the lung and the pancreas. Moreover, numerous conducted in vivo and in vitro studies have clarified that anti-inflammatory properties of citrus flavonoids can be explained via their suppression of synthesis and actions of these proinflammatory mediators, involving nitric oxide (NO), tumor necrosis factor-α (TNF-α), prostaglandins-E2 (PGE2), and IL-1β [106]. In parallel with the present study, Chandravanshi and Bhonde [107] and Yoshimatsu et al. [108], who revealed that implantation of MSCs into diabetic mice, markedly repressed inflammation, but the exact mechanism is still not clear.

IL-10 is considered as a critical anti-inflammatory cytokine that crucially prevents autoimmune pathologies, as well as inflammatory illnesses [109]. In our recent study, STZ-induced diabetic rats showed an apparent decline in pancreatic tissue IL-10 mRNA expression, as clarified by Li et al. [110]. However, the diabetic rats treated with hesperetin and BM-MSCs exhibited a significant increase in the pancreatic tissue mRNA expression of IL-10 compared to animals administered STZ alone. These results run parallel with Comalada et al. [111] and Crouvezier et al. [112], who showed that certain polyphenols, such as quercetin and catechins, may trigger the balance between the pro- and anti-inflammatory mediators’ production, and they improve IL-10 generation, while they suppress TNF-α and IL-1β release. These consequences are consistent with Abdi et al. [113] and Maggini et al. [97], who showed that MSCs augment IL-10 production, thus participating in the noted suppression of NF-κB and leukocyte endothelial interactions. Besides, Li et al. [110] recorded that MSC treatment upregulated anti-inflammatory cytokine IL-10 in STZ-induced diabetic rats.

Taking the effects on the inflammatory mediators, proinflammatory cytokines, and anti-inflammatory cytokines in the present studies and previous studies together, it can be concluded that hesperetin and BM-MSCs have anti-inflammatory potentials, which could have a role in the improvements of the pancreatic islets’ pathological signs, resulting in the increase in the number of β-cells and the insulin secretory response. This, in turn, leads to an increase in blood insulin levels and improvement in the glycemic state after treatment with hesperetin and BM-MSCs.

Regulation of apoptosis occurs via several pro-and anti-apoptotic factors. The apoptotic (e.g., Bax and P53) and anti-apoptotic genes (e.g., Bcl-2) are generally included in proliferation and organized death of cells [114]. The P53 protein plays a vital role in the management of apoptosis, repair of damaged DNA, and cell progress [115,116]. Bcl-2 family proteins are specific for the anti-apoptotic activity and neutralizing pro-apoptotic agents [117]. In the present study, the STZ-induced diabetic rats displayed a significant increase in P53, while they displayed a significant decrease in Bcl-2 mRNA expression levels in pancreatic tissues. These findings are in accordance with El-Kholy et al. [118], who reported that STZ could induce pancreatic β-cell apoptosis in diabetic rats, accompanied with elevated pro-apoptotic protein, p53 and decreased anti-apoptotic protein (Bcl-2) expression, as previously reported by Haidara et al. [119] and Cipollone et al. [95]. The hesperetin-treated diabetic rats exhibited a significant downregulation of P53 and a significant upregulation of Bcl-2 expression compared to those administered STZ alone. This could be attributed the ability of flavonoids to promote the expression of anti-apoptotic genes (e.g., Bcl-2). Therefore, they retain the survival of pancreatic beta cells, as elucidated by Bhattacharya et al. [120], Lee et al. [121], Zhang et al. [122], and Carrasco-Pozo et al. [123]. The BM-MSCs and hesperetin/BM-MSCs-treated diabetic rats showed significant decrease in P53 and increase in Bcl2 expressions compared to animals administered STZ alone. Thus, both BM-MSCs and hesperetin have anti-apoptotic potential in STZ-induced diabetic rats.

In conclusion, BM-MSCs, hesperetin, and their combination have potent antihyperglycemic effects in STZ-induced diabetic Wistar rats; the combinatory effects were the most potent. The antihyperglycemic effects may be secondary to the improvements in the β-cells’ function, thus increasing the insulin secretory response and decreasing hepatic glucose production. The improvement in the pancreatic islets histological architecture and β-cells’ function and histological integrity may be mediated via the suppressive effects of hesperetin and BM-MSCs on inflammation, apoptosis, and oxidative stress, as well as enhancing the antioxidant defense system. However, further investigations are required to study, in more detail, the molecular mechanisms and pathways of the combinatory effects of BM-MSCs and hesperetin in the treatment of DM. Moreover, clinical studies are also required to evaluate the benefits and safety of the mixture of hesperetin and BM-MSCs in diabetic human beings in advance of utilizing these agents as medications in future.

## 4. Materials and Methods

### 4.1. Chemicals

Both STZ and hesperetin were acquired from Sigma Chemical Co. (St. Louis, MO, USA). Dulbecco’s modified Eagle’s medium (DMEM), penicillin/streptomycin solution, trypsin/ethylene diamine tetra acetic acid (EDTA), and fetal bovine serum (FBS) were obtained from Lonza, Antwerp, Belgium. Sodium hydrogen carbonate was obtained from LOBA Chemie, Mumbai, Maharashtra, India. Culture flasks and culture consumables were obtained from Greiner Bio-One (Frickenhausen, Germany). All other reagents utilized in the experiments were of analytical quality and highly pure.

### 4.2. Preparation of Complete Culture Medium

The complete cultured medium was prepared by adding 10% FBS, 1% Penicillin-Streptomycin solution, and 0.36% sodium hydrogen carbonate to 89% DMEM [124] with some modifications.

### 4.3. Isolation, Culture and Proliferation of BM-MSCs

Isolation of BM-MSCs occur via collection of marrow cells from BM of femurs and tibiae of male Wistar rats (4–6 weeks old), according to the instructions of Chaudhary and Rath [125], with some modifications. After decapitation of rats by cervical dislocation and sterilization of the external body surface by 70% (vol/vol) ethanol, femurs and tibiae were dissected, and the associated connective tissues were removed. Using sterile scissors under laminar flow, the epiphysis of each bone was cut, and then cells of BM were harvested in a 15 mL sterile Falcon tube by rinsing with DMEM and centrifugation of cells at 3000 rpm for 5 min at room temperature. Washing and resuspension of the pellet was performed in DMEM supplemented with FBS, sodium hydrogen carbonate, and antibiotic/antimycotic solution in ratios of 15%, 0.36%, and 1%, respectively. The adhering cells were washed twice with pre-warmed (37 °C) 1 × PBS, and then they were trypsinized with 1.0 mL to 2.0 mL of pre-warmed 0.25% trypsin/1 mM EDTA at 37 °C for 2–3 min. Trypsin action was inhibited by adding 2–3 mL complete culturing medium, and cells were collected, centrifuged at 3000 rpm for 5 min in a 15 mL sterile Falcon tube, and then counted, and the viability of cells was determined via trypan blue staining [126] and examination by the inverted microscope to detect viable cells and dead cells. The identification of BM-MSCs was performed according to our previous publication [19].

### 4.4. Experimental Animals

In our study, adult male Wistar rats weighing 100 gm–120 gm were acquired from VACSERA CO. (the Egyptian Company for the production of serums, vaccines, and medicines) Helwan, Egypt, and used for the experimental model. Rats were monitored for one week to rule out any contaminations or infections. The rats were fed on a normal diet and water ad libitum and housed in polypropylene cages with adequate ventilation, at room temperature (RT; 25 ± 5 °C), with a 12 h light /dark cycle. All procedures were carried out in compliance with the recommendations of the Institutional Animal Ethics Committee in Faculty of Science, Beni-Suef University, Egypt (Ethical Approval Number: BSU/FS/2017/17).

### 4.5. Induction of T1DM

After 7 days of adaptation, T1DM was experimentally motivated in 16 h fasting rats via intraperitoneal injection of a freshly prepared STZ solution (sigma company) dissolved in M citrate buffer (pH 4.5) at a dose of 40 mg/kg body weight [127]. STZ injection causes tremendous pancreatic insulin release, leading to fatal hypoglycemia, so the rats were given 5% glucose solution 2 h after STZ administration for the next 48 h to avoid the hypoglycemia and to minimize the mortality that occurs during this period until sustained hyperglycemia was established. The normal rats were intraperitoneally administered a dose of 2 mL/kg b.wt. citrate buffer (vehicle) (pH 4.5). Ten days of STZ administration later, rats were fasted all night (10–12 h), blood samples were collected from the lateral vein of tail region within 2 h (hr) of oral glucose loading (3 g/kg b.wt.), then glucose levels determined with a glucometer system (GlucoDr. Auto) (blood glucose monitoring system). Animals with 2-h serum glucose level exceeding 200 mg/dl were considered diabetic and used in the experiment. Until the end of the experiment, the rats were fed on their respective chows and diets.

### 4.6. Animal Grouping and Experimental Design

**Scheme 1:**
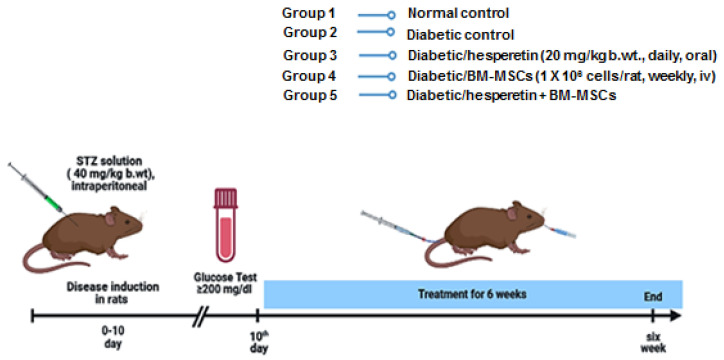
The experimental design and animal grouping, (Created in BioRender.com accessed on 7 February 2023).

After T1DM induction, rats were divided into five groups, and each included six rats (Scheme 1), as follows:

Group I (Normal control): rats were orally given equivalent volumes of the vehicles (citrate buffer, carboxymethylcellulose, or CMC and DMEM).

Group II (Diabetic control): the diabetic rats were orally given the equivalent volume of vehicle (CMC and DMEM).

Group III (Diabetic rats treated with hesperetin): the diabetic rats of this group were orally treated with hesperetin at a dose rate of 20 mg/kg b.wt. dissolved in CMC [128] daily along for six weeks.

Group IV (Diabetic rats treated with BM-MSCs): the diabetic rats of this group were intravenously treated with BM-MSCs at a dose level of 1 × 10^6^ cells/rat weekly for six weeks [28].

Group V (Diabetic rats treated with hesperetin and BM-MSCs): the diabetic rats of this group were treated with hesperetin orally and with BM-MSCs intravenously for six weeks, respectively.

A day prior sacrifice, an oral glucose tolerance test (OGTT) for overnight-fasted rats was performed after oral glucose loading (3 g/kg b.wt.).

### 4.7. Oral Glucose Tolerance Test (OGTT)

OGTT was conducted on all rats of the experiments 24 h before decapitation. Blood specimens were obtained from the lateral tail vein of 10–12 h fasted rats, and blood glucose level measured utilizing a glucometer and GlucoDr.auto strips (Allmedicus, Co, Ltd., Anyang-si, Republic of Korea). The sample of blood were taken at 1, 2, and 3 h following the oral injection of glucose solution (3 g/kg b.w.).

### 4.8. Blood and Tissue Sampling

By the end of the experiment, rats were starved overnight before blood was drawn from the jugular vein using diethyl ether anesthesia and allowed to coagulate at RT. The samples were centrifuged at 3000 rpm for 15 min to separate serum, which was stored at −20 °C, pending its use for the detection of insulin, C-peptide, and fructosamine levels. The liver and pancreas were quickly excised from the dissected rats. A yield of 10% liver homogenate (*w*/*v*) (1-g frozen liver tissue homogenized in 10 mL 0.9% NaCl) was gained, then, it was centrifuged at 3000 round per minute (r.p.m). One part of the pancreas tissue was stored at −70 °C until its use for mRNA determination of NF-κB, IL-1β, IL-10, P53, and Bcl-2 by real time (qRT)-PCR reaction. The other pancreas part was fixed in 10% formalin solution for further histology and immunohistochemistry study.

### 4.9. Detection of Biochemical Parameters

Serum fructosamine level was assayed, as stated by the procedure by Baker et al. [129], with a kit obtained from Reactivos Spinreact Company (Santa Coloma, 7 E-17176 Sant Esteve De Bas, Spain). The glycogen quantity of the liver was assayed via Seifter et al.’s [130] procedure. Activities of liver GP and G6Pase were measured according to the methods of Begum et al. [131] and Stallman and Hers [132], respectively. The liberated inorganic phosphate by G6Pase and GP was determined, as stated by the method of EL-Merzabani et al. [133], using reagents acquired from Biodiagnostic company (Cairo, Egypt).

### 4.10. Determination of Hepatic Oxidative Stress

Determination of lipid peroxidation (LPO), reduced glutathione (GSH) level, glutathione peroxidase (GPx) activity, glutathione reductase (GR) activity, and glutathione-s-transferase (GSTase) activities in hepatic tissues were determined, depending on the principles of Ohkawa et al. (1979), Beutler et al. (1963), Paglia and Valentine (1967), Habig and Jakoby (1974), and Goldberg and Spooner (1983) [134,135,136,137,138], using a reagent kit obtained from Biodiagnostic (Cairo, Egypt).

### 4.11. Enzyme-Linked Immunosorbent Assay (ELISA)

The serum C-peptide, insulin, TNF-α, and IL-4 concentrations were measured by using an ELISA Kit purchased from MyBioSource (Southern California, San Diego, CA, USA), as stated by the instructions of the manufacturer. IL-17 was detected using ELISA kits purchased from CUSABIO (Houston, TX, USA).

### 4.12. Real-Time qPCR Assay for mRNA Level of NF-κB, IL-1β, IL-10, P53 and Bcl-2

The first step is isolation of total RNA from pancreatic tissues, utilizing Qiagen tissue extraction kit (Qiagen, Valencia, CA, USA), as stated by the manufacturer’s instructions. The purity ratio (A260/A280) and the concentration of RNA were determined using dual wavelength Spectrophotometer (Beckman-Coulter, Brea, CA, USA). The second step is reverse transcription of total mRNA into single stranded cDNA, according to the cDNA reverse transcription kit (Fermentas, Thermo Fisher Scientific, Waltham, MA, USA). The third step is quantification of mRNA accumulation of target gene using the Applied Biosystem with software version 3.1 (StepOne™, CA, USA) with SYBR Green dye binding to PCR product via fluorescence PCR using β-actin as a reference. The assay genes were NF-κB (forward 5′- CATTGAGGTGTATTTCACGG -3′, reverse 5′- GGCAAGTGGCCATTGTGTTC -3′), IL-1β (forward 5′- CAC CTT CTT TTC CTT CAT CTT TG -3′, reverse 5′- GTC GTT GCT TGT CTC TCC TTG TA -3′), IL-10 (forward 5′- TGCCAAGCCTTGTCAGAAATGATCAAG -3′, reverse 5′- GTATCCAGAGGGTCTTCAGCTTCTCTC -3′), P53 (forward 5′- CGCAAAAGAAGAAGCCACTA -3′, reverse 5′- TCCACTCTGGGCATCCTT -3′), Bcl-2 (forward 5′- CTACGAGTGGGATGCTGGAGG -3′, reverse 5′- GTCAGATGGACACATGGTG -3′), and β-actin (forward 5′-ATCACCATCTTCCAGGAGCG -3′, reverse 5′-CCTGCTTCACCACCTTCTTG-3′). Lower Ct value indicated higher amounts of PCR products. In addition to profiling all samples for the target sequences, samples were also profiled for β-actin expression (a reference). For the amplification reaction in each well, a Ct was observed in the exponential phase of amplification, and the relative quantification (RQ) levels were achieved using standard curves for both target and endogenous control samples. Relative transcript abundance of a gene is expressed as 2-ΔΔCt values (ΔCt = Cttarget—Ct reference).

### 4.13. Histological Preparations

To conduct histological examination of pancreas, it was immediately excised from each rat after sacrifice and dissection. After that, the pancreas was fixed in 10% neutral buffered formalin and sent to the Histopathology Department, Cancer Institute, Egypt, for preparation. The pancreas was stained with modified aldehyde fuchsin method, 5–6 µm thick sections [139].

### 4.14. Immunohistochemistry Assay of Insulin in Pancreatic Islets

Anti-insulin immunolocalization was performed on 5–6 m thickness sections and stained with the streptavidin–biotin–peroxidase staining procedure [140].

### 4.15. Statistical Analysis

Data from rats in each group were represented as mean ± standard error (SE). Statistics were conducted by one-way analysis of variance (ANOVA) followed by Duncan’s multiple range test [141] using the Statistical Package of Social Sciences (SPSS) program, version 22 (IBM Corp., Armonk, NY, USA). *p* < 0.05, which was taken to specify a significant difference between groups values of probability (*p* > 0.05), which were considered statistically non-significant, while *p* < 0.05 values were considered significant.

## Figures and Tables

**Table 1:** Blood glucose levels (fasting and 2-h post oral glucose loading) in diabetic control and diabetic treated rats 10 days post-STZ injection and at the end of the experiment.

Groups	Glucose Level 10 Days Post-STZ Injection (Pre-Treatment)		Glucose Level at the End of the Experiment (Post-Treatment)	
	Fasting (mg/dL)	2 h (mg/dL)	Fasting (mg/dL)	2 h (mg/dL)
**Diabetic control**	208.91 ± 21.74	459.17 ± 13.16	361.83 ± 36.97 ^#^	490.67 ± 67.55
**Diabetic + hesperetin**	212.60 ± 15.23	489.605 ± 66.05	187.63 ± 29.30 *	189.50 ± 13.52 *^,#^
**Diabetic + BM-MSCs**	225.85 ± 16.41	457.20 ± 74.04	225.38 ± 31.63 *^,$^	226.00 ± 52.38 *^,#^
**Diabetic + hesperetin and BM-MSCs**	221.60 ± 18.99	473.33 ± 78.91	129.3 ± 21.07 *	133.83 ± 9.45 *^,#^

Data are expressed as mean ± standard error (SE). The number of animals in each group is six. * *p* < 0.05 compared with diabetic control group at the same period; ^$^ *p* < 0.05 compared with diabetic group treated with the combination of hesperetin and BM-MSCs; ^#^ *p* < 0.05 compared with their corresponding values at pre-treatment period (10 days post-STZ injection).

**Table 2:** Effects of hesperetin and BM-MSCs on serum fructosamine, insulin, and C-peptide levels of STZ-induced diabetic rats.

Groups	Fructosamine (mmole/L)	Insulin (ng/mL)	C-Peptide (ng/mL)
**Normal**	0.462 ± 0.037	2.50 ± 0.15	6.64 ± 0.27
**Diabetic control**	1.167 ± 0.062 ^†^	1.21 ± 0.07 ^†^	2.72 ± 0.21 ^†^
**Diabetic + hesperetin**	0.562 ± 0.032 *	2.25 ± 0.09 *	5.08 ± 0.27 *
**Diabetic + BM-MSCs**	0.547 ± 0.014 *	2.13 ± 0.09 *	4.75 ± 0.02 *^,$^
**Diabetic + hesperetin and BM-MSCs**	0.565 ± 0.029 *	2.01 ± 0.03 *	5.46 ± 0.15 *

Data are expressed as mean ± standard error (SE). The number of animals in each group is six. ^†^ *p* < 0.05 compared with normal group; * *p* < 0.05 compared with diabetic control group; ^$^ *p* < 0.05 compared with diabetic group treated with the combination of hesperetin and BM-MSCs.

**Table 3:** Effects of hesperetin and BM-MSCs on glycogen, G6PAase, and GP levels in liver of STZ-induced diabetic rats.

Groups	Liver Glycogen (mg Glycogen/g Liver)	Liver G6Pase (mg Pi Liberated/g Tissue/h)	Liver GP (mg Piliberated/g Tissue/h)
**Normal**	11.44 ± 1.44	0.37 ± 0.01	0.36 ± 0.02
**Diabetic control**	0.50 ± 0.07 ^†^	0.76 ± 0.06 †	0.97 ± 0.04 ^†^
**Diabetic + hesperetin**	2.10 ± 0.33 *^,$^	0.43 ± 0.01 *	0.57 ± 0.06 *^,$^
**Diabetic + BM-MSCs**	9.00 ± 0.64 *^,$^	0.43 ± 0.01 *	0.67 ± 0.07 *
**Diabetic + hesperetin and BM-MSCs**	4.25 ± 0.29 *	0.46 ± 0.01 *	0.76 ± 0.05 *

Data are expressed as mean ± standard error (SE). The number of animals in each group is six. ^†^ *p* < 0.05 compared with normal group; * *p* < 0.05 compared with diabetic control group; ^$^ *p* < 0.05 compared with diabetic group treated with the combination of hesperetin and BM-MSCs.

**Table 4:** Effects of hesperetin and BM-MSCs on LPO, GSH, and GPx activities in the liver of STZ-induced diabetic rats.

Groups	LPO (nmole MDA/100 mg Tissue)	GSH (nmole/100 mg Tissue)	GPx (U/g Tissue)
**Normal**	26.73 ± 0.59	57.27 ± 1.14	48.20 ± 4.40
**Diabetic control**	142.77 ± 14.65 ^†^	24.83 ± 1.01 ^†^	17.72 ± 0.96 ^†^
**Diabetic + hesperetin**	90.42 ± 3.92 *	34.77 ± 1.79 *	20.09 ± 0.71 ^$^
**Diabetic + BM-MSCs**	64.78 ± 4.44 *^,$^	50.20 ± 0.99 *^,$^	28.95 ± 1.21 *
**Diabetic + hesperetin and BM-MSCs**	76.95 ± 6.95 *	34.37 ± 1.96 *	26.46 ± 0.75 *

Data are expressed as mean ± standard error (SE). The number of animals in each group is six. ^†^ *p* < 0.05 compared with normal group; * *p* < 0.05 compared with diabetic control group; ^$^ *p* < 0.05 compared with diabetic group treated with the combination of hesperetin and BM-MSCs. LPO, lipid peroxide; GSH, reduced glutathione; GPx, glutathione peroxidase.

**Table 5:** Effects of hesperetin and BM-MSCs on GST and GR activities in the liver of STZ-induced diabetic rats.

Groups	GST (U/100 mg Tissue)	GR (mU/100 mg Tissue)
**Normal**	116.83 ± 3.60	14 ± 0.73
**Diabetic control**	62.20 ± 1.93 ^†^	5.5 ± 0.55 ^†^
**Diabetic + hesperetin**	92.43 ± 4.77 *^,$^	20.17 ± 0.83 *
**Diabetic + BM-MSCs**	82.88 ± 2.99 *^,$^	19.67 ± 1.76 *
**Diabetic + hesperetin and BM-MSCs**	69.42 ± 2.66 ^†^	22.17 ± 0.95 *

Data are expressed as mean ± standard error (SE). The number of animals in each group is six. ^†^ *p* < 0.05 compared with normal group; * *p* < 0.05 compared with diabetic control group; ^$^ *p* < 0.05 compared with diabetic group treated with the combination of hesperetin and BM-MSCs.

**Table 6:** Effects of hesperetin and BM-MSCs on pancreatic tissues of NF-κB, IL-1β, and IL-10 mRNA gene expressions of STZ-administered rats.

Groups	NF-κB (Fold Change, Relative to Normal)	IL-1β (Fold Change, Relative to Normal)	IL-10 (Fold Change, Relative to Normal)
**Normal**	1.01 ± 0.002	1.01 ± 0.002	1.01 ± 0.004
**Diabetic control**	5.00 ± 0.1 1 ^†^	6.65 ± 0.09 ^†^	0.25 ± 0.03 ^†^
**Diabetic + hesperetin**	1.63 ± 0.16 *^,$^	2.45 ± 0.05 *^,$^	0.65 ± 0.03 *^,$^
**Diabetic + BM-MSCs**	1.45 ± 0.05 *^,$^	2.24 ± 0.06 *	0.76 ± 0.01 *
**Diabetic + hesperetin and BM-MSCs**	2.05 ± 0.01 *	2.10 ± 0.22 *	0.72 ± 0.01*

Data are expressed as mean ± standard error (SE). The number of animals in each group is six. ^†^ *p* < 0.05 compared with normal group; * *p* < 0.05 compared with diabetic control group; ^$^ *p* < 0.05 compared with diabetic group treated with the combination of hesperetin and BM-MSCs.

**Table 7:** Effects of hesperetin and BM-MSCs on serum TNF-α, IL-17 and IL-4 levels in STZ-induced diabetic rats.

Groups	TNF-α (pg/mL)	IL-17 (pg/mL)	IL-4 (pg/mL)
**Normal**	14.73 ± 0.47	42.57 ± 1.42	120.50 ± 2.80
**Diabetic control**	106.12 ± 3.50 ^†^	122.35 ± 2.10 ^†^	40.03 ± 3.66 ^†^
**Diabetic + hesperetin**	49.27 ± 2.56 *^,$^	68.13 ± 1.86 *^,$^	90.33 ± 3.65 *
**Diabetic + BM-MSCs**	43.40 ± 2.35 *	66.00 ± 6.68 *^,$^	95.10 ± 4.83 *
**Diabetic + hesperetin and BM-MSCs**	39.33 ± 5.12 *	52.20 ± 4.11 *	96.77 ± 5.93 *

Data are expressed as mean ± standard error (SE). The number of animals in each group is six. ^†^ *p* < 0.05 compared with normal group; * *p* < 0.05 compared with diabetic control group; ^$^ *p* < 0.05 compared with diabetic group treated with the combination of hesperetin and BM-MSCs.

**Table 8:** Effects of hesperetin and BM-MSCs on pancreatic tissue P53 and Bcl-2 mRNA expressions of STZ-administered albino rats.

Groups	P53 (Fold Change, Relative to Normal Control Rats)	Bcl-2 (Fold Change, Relative to Normal Control Rats)
**Normal**	1.02 ± 0.01	1.01 ± 0.002
**Diabetic control**	6.77 ± 0.1 ^†^	0.23 ± 0.004 ^†^
**Diabetic + hesperetin**	3.21 ± 0.07 *^,$^	0.56 ± 0.03 *^,$^
**Diabetic + BM-MSCs**	2.95 ± 0.09 *	0.57 ± 0.01 *^,$^
**Diabetic + hesperetin and BM-MSCs**	2.65 ± 0.31 *	0.65 ± 0.01 *

Data are expressed as mean ± standard error (SE). The number of animals in each group is six. ^†^ *p* < 0.05 compared with normal group; * *p* < 0.05 compared with diabetic control group; ^$^ *p* < 0.05 compared with diabetic group treated with the combination of hesperetin and BM-MSCs.

**Table 9:** Effects of hesperetin and BM-MSCs on histopathological lesion scores of islet cells vacuolation and necrosis in STZ-induced diabetic rats.

	Histopathological Lesion Score	
Groups	Vacuolations	Necrosis
**Normal**	−	−
**Diabetic control**	+++	+++
**Diabetic + hesperetin**	+	−
**Diabetic + BM-MSCs**	++	+
**Diabetic + hesperetin and BM-MSCs**	−	−

The degrees of histopathological scores of vacuolations and necrosis. − absent, + mild, ++ moderate, and +++ severe.

## Data Availability

Data is contained within the article.
